# Intralesional bevacizumab as an adjuvant approach to recurrent respiratory papillomatosis: A prospective study on different treatment protocols

**DOI:** 10.1371/journal.pone.0342749

**Published:** 2026-02-17

**Authors:** Hanna Klimza, Bartosz Zakrzewski, Andrzej Porębski, Małgorzata Wierzbicka

**Affiliations:** 1 Otolaryngology Department with the Paediatric Otolaryngology Subunit, Regional Specialist Hospital, Wroclaw, Poland; 2 Faculty of Law and Administration, Jagiellonian University, Kraków, Poland; 3 Department of Surgical Clinical Sciences, Faculty of Medicine, Wroclaw University of Science and Technology, Wroclaw, Poland; 4 Institute of Human Genetics, Polish Academy of Sciences, Poznan, Poland; Universiti Malaya Fakulti Perubatan: University of Malaya Faculty of Medicine, MALAYSIA

## Abstract

**Objectives:**

Adjuvant intralesional administration of bevacizumab has shown promising effects in the management of recurrent respiratory papillomatosis (RRP). However, the optimal therapeutic parameters remain undefined. This prospective study aimed to compare the efficacy of two distinct intralesional bevacizumab administration protocols following surgical resection of RRP.

**Methods:**

Between 15/09/2023 and 31/12/2024, adult patients with RRP were enrolled in a prospective research study. Participants were allocated to one of two bevacizumab administration protocols, delivered intralesional during direct microlaryngoscopy and CO₂ laser excision. Disease severity was assessed using the Derkay, Dikkers and Numerical scoring systems, as well as the Voice Handicap Index (VHI), at baseline and at 8-week intervals.

**Results:**

Both treatment regimens were associated with reduction in disease burden and improved voice outcomes. No statistically significant differences in disease control were observed between the two administration protocols.

**Conclusion:**

This study indicates that both dosing regimens of bevacizumab were effective as an adjunctive therapy of recurrent respiratory papillomatosis. The higher intralesional doses of bevacizumab are relatively safe and well tolerated in adult patients with laryngeal RRP. Further studies with extended follow-up are warranted to define long-term efficacy and optimal dosing strategies.

## Introduction

Recurrent respiratory papillomatosis (RRP) is a rare but clinically burdensome benign airway disease, characterized by unpredictable recurrences that compromise voice, airway patency, and quality of life. Current management relies almost exclusively on repeated surgical debulking, a palliative strategy associated with cumulative morbidity and persistent risk of airway compromise. This creates a clear and unmet need for adjuvant treatments that can durably reduce disease burden and surgical dependency.

Bevacizumab, a monoclonal antibody targeting vascular endothelial growth factor (VEGF), has emerged as a promising intralesional therapy in RRP [1,2]. In routine practice, bevacizumab administration has largely been guided empirically by laryngoscopic appearance and patient preference rather than standardized protocols. Several small series have reported reductions in papilloma volume and lengthening of intersurgical intervals, including a cohort of nineteen adults treated with KTP laser ablation and sublesional bevacizumab, but dosing and scheduling have remained empirical and non‑standardized [3–6]. Robust, protocol‑driven data on optimal intralesional regimens are therefore lacking.

The present prospective study was designed to address this gap by directly comparing two predefined intralesional bevacizumab protocols in adult RRP. **By applying standardised dosing schedules and prospectively assessing the achievement of sustained remission for at least six months as the primary endpoint, this trial aims to provide clinically actionable evidence to guide rational, protocol‑based use of bevacizumab in RRP management.**

## Materials and methods

### Study population and design

Between 15/09/2023 and 31/12/2024, sixteen adult patients with severe recurrent respiratory papillomatosis (RRP) were enrolled at the Research and Development Centre, Wroclaw Regional Specialist Hospital. Ethical approval was obtained from the institutional review board. All patients provided written informed consent for participation in the prospective research study and off-label intralesional bevacizumab administration. Patients were randomly allocated to one of two treatment arms: **MLS-3** (n = 9) and **MLS-5** (n = 7). All microlaryngeal surgeries were performed under general anaesthesia.

Prior to surgery, all patients underwent flexible transnasal videoendoscopy (Olympus Medical Systems, Tokyo, Japan) using both white light imaging (WLI) and narrow-band imaging (NBI). Papillomatous lesions typically appeared as raspberry-like sessile or exophytic growths on WLI, with intrapapillary capillary loops displaying wide-angled turning points on NBI. To standardise disease assessment, three validated scoring systems (Numerical [7], Derkay [8], Dikkers [9]) were applied. Across the cohort, 88 assessments were performed at baseline, after each bevacizumab injection, and at six months following the final intervention, which was considered the definitive evaluation of treatment response. Each patient underwent 4–8 evaluations depending on group allocation. In addition, voice function was assessed using the Voice Handicap Index (VHI) during assessments.

### Ethical approval

All procedures performed in studies involving human participants were in accordance with the ethical standards of the institutional and national research committee, as well as with the 1964 Helsinki Declaration and its later amendments or comparable ethical standards. The Bioethics Committee of Lower Silesian Medical Chamber approved the study protocol (approval number 10/BOBD/2023)

### Inclusion criteria

Eligible patients were ≥18 years of age and presented with either juvenile-onset (JORRP) or adult-onset RRP (AORRP). Indications for enrolment included requirement for >4 surgical interventions per year, rapid recurrence (<3 months) following resection, Derkay severity score >5, bilateral or multifocal disease, and/or lower respiratory tract spread. Exclusion criteria included prior intralesional injections, inability to comply with follow-up, or refusal of off-label therapy.

### Treatment protocols

The MLS-3 group underwent three microlaryngeal surgeries at 8-week intervals. During each procedure, bevacizumab (20 mg/ml) was injected intralesionally, with 0.25 ml delivered to each of four predefined anatomical sites of the larynx (right vocal fold, left vocal fold, subglottis, epiglottis).

The MLS-5 group underwent five microlaryngeal surgeries at 8-week intervals, with bevacizumab administered at a lower concentration (10 mg/ml) but in an identical site-specific distribution (0.25 ml per site).

Treatment response at the six-month follow-up was categorised as:

- Complete response: score of 0 in all three systems.
- Partial response: Derkay 1–2 points in one site, Numerical score limited to 1–2 sites, and/or Dikkers 1–2 points.
- No response: any score exceeding the above thresholds.

### Bevacizumab administration

During microlaryngeal surgery, bevacizumab was delivered in a strictly standardised manner. For *superficial lesions*, the drug was injected prior to surgical removal. For *exophytic lesions*, excision preceded injection. In *mixed lesions*, both approaches were combined according to the dominant morphology.

All injections were administered with a 25G or finer needle into the subepithelial layer (Reinke’s space) under microscopic or endoscopic guidance, with precise dosing (0.25 ml per site). Care was taken to avoid vascular structures and to monitor tissue response, ensuring prevention of ischaemia. Injection details and perioperative observations were documented for each patient.

### Aims and outcome measures

The primary aim was to compare treatment response between the two study groups, MLS-3 and MLS-5. The main outcomes included changes in three validated disease severity scores (the Dikkers, Derkay, and Numerical RRP scales). To summarise the Derkay scale, the sum was used, and to summarise the Numerical RRP scale, the number of segments was counted. The values obtained using NBI were used as they may be more accurate than the values obtained using WLI [10]. The additional outcomes included VHI general score and VHI subscales. The secondary aim was the assessment of the correlation between three scoring systems and the VHI.

## Results

To check the overall effect of treatment, one-sample *t*-tests were used to test the significance of the difference from 0 between the first and last patient’s measurements in the outcomes considered. A higher value represents a greater decrease, so the greatest possible decreases and values significantly higher than 0 are desired. As the tests showed, highly significant differences were observed in the decrease in the Dikkers, Derkay and Numerical scales (p < 0.001) and in the VHI general score (*p* = 0.006), while significant decreases were observed in the functional VHI (*p* = 0.014) and physical VHI (*p* = 0.021) subscales, see Table 1. The only insignificant decrease was in the emotional VHI (*p* = 0.206). Cohen’s *d* values suggest a very strong treatment effect for changes in the disease severity scores (*d* > 1.4 for each) and a medium-high effect for significant changes in VHI (*d* > 0.6), but with the sample obtained, the estimates of the effects are not very stable (SE Cohen’s *d* from 0.286 to 0.456), so only Cohen’s *d* for the decrease in the disease severity scores should be considered indicative of a strong effect, see Table 1.

**Table 1:** Overall treatment outcomes and *t*-tests of reduction in VHI and the disease severity scores.

Variable	*t*	df	*p*	Mean Difference	Cohen’s *d*	SE Cohen’s *d*
Change in Dikkers	7.766	15	< 0.001	1.938	1.942	0.425
Change in Derkay (Sum)	8.620	15	< 0.001	5.563	2.155	0.456
Change in Numerical (Number)	5.948	15	< 0.001	6.250	1.487	0.363
Change in General VHI	3.258	14	0.006	9.200	0.841	0.300
Change in Functional VHI	2.822	14	0.014	4.333	0.729	0.290
Change in Emotional VHI	1.325	14	0.206	1.933	0.342	0.266
Change in Physical VHI	2.607	14	0.021	3.000	0.673	0.286

No significant differences were found between MLS-3 and MLS-5 groups when the differences in outcomes were compared using independent samples *t*-tests with the Welch correction (each *p*-value > 0.24), see Table 2. None of the *p*-values even came close to the significance threshold. Some differences were observed in the sample only, but they were not consistently structured, see Table 3: some of the differences were in favour of the MLS-3 group and some in favour of the MLS-5 group, which suggests that no pattern can be found, but rather that differences in the sample can be explained by the randomness. Moreover, the independence between MLS-3 vs MLS-5 compared to the categorised treatment outcome was also tested. The differences were insignificant both when comparing the three response levels (none, partial, or complete response) with the MLS groups (*Χ*^2^(1) = 0. 907, *p* = 0.635) and when two response levels (complete vs. other than complete response) were compared with the MLS groups (*Χ*^2^(2) = 0.788, *p* = 0.375). Thus, this study provides no basis for differentiating between the effects achieved by the MLS-3 and MLS-5 groups.

**Table 2:** Comparison between MLS-3 and MLS-5 groups in the reduction of outcome measures using Welch’s *t*-test.

Variable	*t*	df	*p*	Mean Difference	SE Difference
Change in Dikkers	−1.220	11.563	0.247	−2.603	2.134
Change in Derkay (Sum)	0.283	13.962	0.781	0.143	0.505
Change in Numerical (Number)	−0.012	12.638	0.991	−0.016	1.357
Change in General VHI	0.264	12.999	0.796	1.444	5.472
Change in Functional VHI	1.023	11.439	0.328	2.778	2.716
Change in Emotional VHI	0.383	7.989	0.712	1.278	3.335
Change in Physical VHI	−1.099	11.823	0.294	−2.500	2.274

**Table 3:** Group descriptives of the reduction of outcome measures in MLS-3 and MLS-5 groups.

Variable	Group	*N*	Mean	SD	SE	Coeff. of Variation
Change in Dikkers	MLS3	9	5.111	3.756	1.252	0.735
	MLS5	7	7.714	4.572	1.728	0.593
Change in Derkay (Sum)	MLS3	9	2.000	1.118	0.373	0.559
	MLS5	7	1.857	0.900	0.340	0.484
Change in Numerical (Number)	MLS3	9	5.556	2.603	0.868	0.469
	MLS5	7	5.571	2.760	1.043	0.495
Change in General VHI	MLS3	9	9.778	12.833	4.278	1.313
	MLS5	6	8.333	8.359	3.412	1.003
Change in Functional VHI	MLS3	9	5.444	7.265	2.422	1.334
	MLS5	6	2.667	3.011	1.229	1.129
Change in Emotional VHI	MLS3	9	2.444	4.799	1.600	1.963
	MLS5	6	1.167	7.167	2.926	6.143
Change in Physical VHI	MLS3	9	2.000	4.637	1.546	2.318
	MLS5	6	4.500	4.087	1.668	0.908

There were no statistically significant correlations between the change in VHI scores and the change in the disease severity scores (*n* = 15): the Dikkers, Derkay and Numerical RRP scales (the *p*-value in one of the twelve pairs was 0.066, in the others > 0.13), see Table 4. This suggests that the improvement measured by the VHI is at least partially independent of the improvement measured by disease severity scores. In other words, a stronger decline in Dikkers, Derkay, and Numerical scores does not necessarily translate into a stronger decline in the VHI or VHI subscales, and vice versa. Additionally, this result was reinforced by checking the correlation of the VHI and VHI subscales with three disease severity scores on single measurements (*n* = 65) during the study. Again, no statistically significant correlations were found (the *p*-value in one of the twelve pairs was 0.057, in the others > 0.21).

**Table 4:** Correlations of changes in VHI and disease severity scores.

Variable		Change in General VHI	Change in Functional VHI	Change in Emotional VHI	Change in Physical VHI
Change in Dikkers	Pearson’s *r*	−0.103	−0.125	0.266	−0.405
	*p*	0.715	0.656	0.337	0.135
Change in Derkay (Sum)	Pearson’s *r*	0.126	−0.007	0.334	−0.114
	*p*	0.655	0.979	0.224	0.686
Change in Numerical (Number)	Pearson’s *r*	0.129	−0.217	0.487	−0.015
	*p*	0.646	0.438	0.066	0.958

## Discussion

Recurrent respiratory papillomatosis (RRP) remains a disease without a definitive causal therapy, with surgery serving as the mainstay of management. However, repeated interventions within the delicate glottic region carry substantial risks, including scarring, anterior web formation and stenosis, which may result in irreversible voice impairment and reduced quality of life [11]. These concerns have prompted the ongoing search for effective adjuvant therapies [12–14].

Over past decades, several agents including interferon and cidofovir were investigated, but their unfavourable safety profiles and inconsistent efficacy have relegated them to historical interest. More recently, bevacizumab, a monoclonal antibody targeting vascular endothelial growth factor (VEGF), has emerged as the most promising pharmacological adjunct [15]. VEGF is a key driver of papilloma growth, and inhibition of this pathway has demonstrated beneficial effects in both paediatric and adult populations. Bevacizumab may be administered intravenously or intralesional. Intravenous protocols have been associated with excellent disease control, as reported by Best et al., yet they carry the disadvantages of systemic exposure and recurrence following discontinuation [16]. For this reason, intralesional administration remains clinically attractive, combining a favourable safety profile with practicality during microlaryngeal surgery.

The present study addressed a knowledge gap by directly comparing two structured intralesional bevacizumab dosing regimens. Overall, the treatment led to a statistically significant improvement in all outcomes except for the emotional VHI. However, no significant difference in efficacy was observed between the two dosing groups. Importantly, we found that a higher dose administered in fewer treatment sessions was similarly effective and safe, offering a more cost-efficient option for clinical practice. This finding is consistent with previous reports of high-dose sublesional administration without increased risk of local or systemic complications [3].

In response to the lack of standardised approaches, this study provides a detailed description of a lesion‑specific intralesional injection technique. For superficial lesions, bevacizumab was injected into Reinke’s space before excision, creating hydrodissection and aiding both exposure and drug retention. In contrast, for bulky exophytic disease, injection after excision proved technically advantageous by avoiding unnecessary bulging and maintaining surgical control. These technical refinements expand on existing recommendations, aligning injection strategy with lesion morphology.

The study did not find any significant correlations between changes in VHI and the composite values of the disease severity scales, nor between their raw values (resulting from individual measurements). These findings are consistent with a full or partial independence between examined constructs: (1) the VHI and its subscales and (2) the Dikkers, Derkay, and Numerical scales. This suggests that VHI is distinct from other scales and conveys different information. Thus, the VHI and severity scales should be evaluated and considered independently. However, this observation should be interpreted with caution given the limited statistical power resulting from the relatively small sample size.

The study has limitations, including the modest sample size and short follow-up duration. Nevertheless, it benefits from relatively homogeneous treatment groups and assessment using three complementary scoring systems alongside the Voice Handicap Index, strengthening the reliability of our findings. Continued follow-up will be essential to confirm long-term efficacy and safety.

It is imperative to emphasize that no safety issues have been documented with any dosage regimen.

## Conclusions

Both intralesional dosing regimens of bevacizumab were effective and well tolerated in adults with laryngeal RRP. Comparable clinical responses support the use of a higher dose delivered over fewer treatment sessions, which appears both safe and cost-efficient. These results may inform clinical practice and serve as a basis for future studies with larger cohorts and longer follow-up to establish long-term treatment guidelines.

## Clinical relevance

This study provides evidence that intralesional bevacizumab is a safe and effective adjunctive therapy in adult patients with recurrent respiratory papillomatosis. A regimen of higher-dose administration during three treatment sessions achieved outcomes comparable to lower-dose regimens requiring more interventions, offering a more practical and cost-efficient strategy. The lesion-specific injection technique described here may further optimize surgical management by improving exposure, reducing recurrence, and minimizing the risk of laryngeal complications.

## Supporting information

S1 Table Underlying data table supporting the statistical analysis. (XLSX)
